# Electromagnetic Interference Shielding and Physical-Mechanical Characteristics of Rubber Composites Filled with Manganese-Zinc Ferrite and Carbon Black

**DOI:** 10.3390/polym13040616

**Published:** 2021-02-18

**Authors:** Ján Kruželák, Andrea Kvasničáková, Klaudia Hložeková, Rastislav Dosoudil, Marek Gořalík, Ivan Hudec

**Affiliations:** 1 Department of Plastics, Rubber and Fibres, Faculty of Chemical and Food Technology, Slovak University of Technology in Bratislava, 812 37 Bratislava, Slovakia; andrea.kvasnicakova@stuba.sk (A.K.); klaudia.hlozekova@stuba.sk (K.H.); ivan.hudec@stuba.sk (I.H.); 2 Department of Electromagnetic Theory, Faculty of Electrical Engineering and Information Technology, Slovak University of Technology in Bratislava, 812 19 Bratislava, Slovakia; rastislav.dosoudil@stuba.sk; 3 Polymer Centre, Faculty of Technology, Tomas Bata University in Zlín, 760 01 Zlín, Czech Republic; goralik@utb.cz

**Keywords:** electromagnetic interference, absorption shielding, physical-mechanical properties, ferrite, carbon black

## Abstract

In the present work, composite materials were prepared by incorporation of manganese-zinc ferrite, carbon black and combination of ferrite and carbon black into acrylonitrile-butadiene rubber (NBR). For cross-linking of composites, standard sulfur-based curing system was applied. The main goal was to investigate the influence of the fillers on the physical-mechanical properties of composites. Then, the electromagnetic absorption shielding ability was investigated in the frequency range 1 MHz–3 GHz. The results revealed that composites filled with ferrite provide sufficient absorption shielding performance in the tested frequency range. On the other hand, ferrite behaves as an inactive filler and deteriorates the physical-mechanical characteristics of composites. Carbon black reinforces the rubber matrix and contributes to the improvement of physical-mechanical properties. However, composites filled with carbon black are not able to absorb electromagnetic radiation in the given frequency range. Finally, the combination of carbon black and ferrite resulted in the modification of both physical-mechanical characteristics and absorption shielding ability of hybrid composites.

## 1. Introduction

The rapid progress in today’s modern society has been connected with the development of high amount of electronic and communication devices. Each electronic appliance produces electromagnetic radiation, which is emitted into the surrounding. The accumulation of electromagnetic radiation leads to the formation of electromagnetic smog, or electromagnetic interference (EMI), which not only negatively affects the functionality of electronics, e.g., irreparable loss of valuable stored data, signal interruption, etc., but also deteriorates the human health [1,2]. Thus, the development of various types of EMI shielding materials has attracted considerable interest over last decades.

The electromagnetic interference shielding effectiveness can be understood as the sum of reflection, absorption and multiply reflection of EMI [3,4,5]. The basic condition for reflection shielding is the materials electric conductivity, while materials possessing magnetic or electric dipoles are suitable candidates for absorption shielding. The multiply reflection operates via the internal reflections of electromagnetic radiation waves from various in-homogeneities and phase interfaces within the shield [6,7]. From the practical point of view, the shielding by absorption has become the most demanding, as the electromagnetic radiation waves are efficiently absorbed by the shield and not emitted back to the surrounding.

Carbon black (CB) is the most frequently used filler in rubber industry, due to its simple manufacturing, tunable physical properties and relatively low cost. It is used as reinforcing filler in a variety of rubber products such as tires, conveyor belts, footwear, or shock absorbers. Carbon black is produced by incomplete combustion or thermal decomposition of hydrocarbons at high temperatures. Depending on the production process, carbon black can have different particle size and structure. The parallel graphitic layer planes form elementary crystallites having 100–200 carbon atoms. During the production process, the primary particles connect together to form three dimensional structural aggregates or agglomerates. In addition to carbon atoms, carbon black contains small amounts of chemically-combined oxygen and hydrogen groups [8,9].

Ferrites as magnetic substances are a class of technologically very important materials owing to their high permeability, soft magnetic nature, good chemical stability, water and weather resistance, and low price. They have been widely used for magnetic and magnetic-optical recording, in electrical components, transformers, choke coils, memory devices, or noise filters, etc. [10,11]. Ferrites may occur in several crystallographic modifications. In the case of magnetically soft ferrites, the spinel structure is the most preferred. Spinel ferrites are very stable due to their stable crystal structure and they are predominantly ionic [12]. They are composed from tetrahedral and octahedral sites, in which metal ions are trapped with oxygen ions.

The application of carbon black and ferrites into the polymer matrices in order to prepare composites with the effects of EMI shielding has been the subject of various scientific studies [13,14,15,16,17,18,19]. The results have shown that application of both fillers leads to the preparation of polymer composites that can efficiently shield the harmful electromagnetic radiation. However, majority of scientific studies have been oriented on shielding efficiency at high frequencies, mainly in the X-frequency band range (8.2–12.4 GHz) or even higher frequencies. Most of generally used electronic devices and equipment (mobile phones, TV sets, radios, laptops, etc.) emit electromagnetic radiation in frequency range 0.6–3 GHz. Therefore, for the protection of such equipment, it is very important to know the shielding efficiency at low frequencies. In the present work, manganese-zinc ferrite as magnetic filler and carbon black as the commonly used carbon-based filler were solely used for the fabrication of composite materials with the utilization of NBR as the rubber matrix. The physical-mechanical characteristics and EMI absorption shielding efficiency of the composites were investigated at low frequencies. Then, carbon black was combined with ferrite and incorporated into the rubber matrix with the aim to modify both the tensile characteristics and absorption shielding performance.

## 2. Experimental

### 2.1. Materials

Acrylonitrile-butadiene rubber (NBR, type SKN 3345, content of acrylonitrile 31–35%) supplied from Sibur International (Moscow, Russia) was used as rubber matrix. Carbon black (CB, type Vulcan XC72, multi-purpose, specialty carbon black with medium electrical conductivity, moderate process-ability, low sulfur, and grit levels) was supplied from Continental Carbon Company (Houston, Texas, USA). Manganese-zinc ferrite MnZn provided by Epcos s.r.o (Šumperk, Czech Republic) was used as magnetic soft ferrite filler. The structural and physical characteristics of the magnetic filler are presented in Table 1. For cross-linking of composites, a standard sulfur based curing system consisting of stearic acid and zinc oxide (Slovlak, Košeca, Slovakia) as activators, accelerator N-cyclohexyl-2-benzothiazole sulfenamide (CBS, Duslo, Šaľa, Slovakia) and sulfur (Siarkopol, Tarnobrzeg, Poland) was used.

### 2.2. Methods

#### 2.2.1. Preparation and Curing of Rubber Compounds

In the current work, three types of rubber formulations were prepared and tested. The amounts of curing additives were kept constant in all rubber compounds and there was a change only in the type and amount of the filler. Manganese-zinc ferrite in a concentration scale ranging from 100 to 500 phr was used as filler in the first type of composites. Our previous experiments have shown that at least 200 or 300 phr of ferrite is necessary to achieve the acceptable absorption shielding performance of rubber composites. Carbon black was incorporated in the second type of composites in the amount ranging from 2.5 to 25 phr. The usual amount of carbon black in generally used rubber articles ranges from 10 to 30 phr, therefore, the choice of CB concertation scale was adopted to above mentioned concentration range. In the third composite types, the content of CB was kept on constant level—20 phr and the amount of manganese-zinc ferrite was changed from 100 to 500 phr. The detailed compositions of rubber composites are summarized in Table 2, Table 3 and Table 4.

The compounding of additives was carried out in two steps using an industrial kneading machine Buzuluk (Buzuluk Inc., Komárov, Czech Republic) and a laboratory kneading equipment Brabender (Brabender GmbH & Co. KG, Duisburg, Germany). The speed of the rotor was set up to 50 rpm and the kneading chamber was heated to 90 °C. For the preparation of rubber compounds with carbon black, the NBR/CB batch containing 25 phr of CB was first fabricated using the kneading machine Buzuluk. Rubber was added into the chamber and after 1 min of plasticization, CB was introduced and the whole mixture was compounded for the next 4 min. Then, the rubber compound was cooled and shaped into a rubber sheet using a two-roll calender. For the detailed dispersion of CB within the rubber matrix, the rubber compound was again added into the chamber of kneading machine and was compounded for the next 3 min at 90 °C and 50 rpm followed by final shaping in the two-roll calender. Laboratory mixing equipment was then used for the preparation of composites containing different amount of CB. The procedure consisted of two steps at 90 °C and 50 rpm. First, NBR was put into the chamber, NBR/CB batch was subsequently added. The compounding of ingredients took next 2.5 min. Stearic acid and ZnO were then added with subsequent mixing for 6.5 min. CBS and sulfur were introduced in the second step (4 min, 90 °C, 50 rpm). Finally, the two-roll calender was introduced to form the fabricated compounds into thin rubber sheets.

In the case of composites filled with ferrite, NBR was first plasticated for 2.5 min, subsequently activators were added and after next two 2 min filler was applied. The total time of first step mixing was 9 min at 90 °C and 50 rpm. In the second step (4 min, 90 °C, 50 rpm), the accelerator CBS and sulfur were introduced. Finally, the composites were homogenized in two-roll calender.

The preparation procedure of composites filled with carbon black and magnetic filler proceeded in the same way as in the previous case, but NBR/CB batch was first compounded with pure NBR to reduce the amount of CB to 20 phr. Then, the compounding procedure followed the same conditions.

The curing process of composites was performed at 160 °C for the optimum curing time under a pressure of 15 MPa by using a hydraulic press Fontijne (Fontijne, Vlaardingen, Holland). Finally, thin sheets (width 15 × 15 cm, thickness 2 mm) of cured rubber compounds were obtained.

#### 2.2.2. Investigation of Physical-Mechanical Characteristics

The tensile properties of composites were evaluated by using Zwick Roell/Z 2.5 appliance (Zwick Roell Group, Ulm, Germany). The cross-head speed of the measuring device was set up to 500 mm/min and the tests were carried out in compliance with the valid technical standards. Dumbbell-shaped test specimens (thickness 2 mm, length 80 mm, width 6.4 mm) were used for measurements.

#### 2.2.3. Investigation of Shielding Characteristics

The frequency dependences of complex permeability and complex permittivity of the fabricated composite materials were measured in the range 1 MHz–3 GHz by means of a combined impedance/transmission line method using a vector network analyzer Keysight E5063A (Keysight Technologies, Santa Rosa, California, USA). The DC electrical conductivity was determined using standard two probe method. Based on frequency responses of material parameters, the monolayer EM-wave absorption characteristics (return loss *RL*, matching thickness *d*_m_, matching frequency f_m_, bandwidth ∆f for *RL* ≤ −10 dB and *RL* ≤ −20 dB, and the minimum of return loss *RL*_min_) were computed from return loss *RL*. It is defined as follows:

(1) $$RL=20\log\left|\frac{Z_{in}-Z_{0}}{Z_{in}+{\;Z}_{0}}\right|$$

where:

(2) $$Z_{in}=\;\sqrt{\frac{\mu \;}{\varepsilon }}\tan h\left|i\frac{\omega d}{c}\left(\mu \times \varepsilon \right)\right|$$

is the normalized value of input complex impedance of the absorber, ω is the angular frequency, *d* is the thickness of the monolayer absorber (backed by a metal sheet), and *c* is the velocity of light in free space. The composite absorbs the maximum of the energy if *Z*_*in*_ = 1, which is reached at a matching thickness *d* = *d*_m_, matching frequency f = f_m_, and minimum return loss *RL* = *RL*_min_.

#### 2.2.4. Microscopic Analysis

The surface morphology and microstructure of composites were observed using a JEOL JSM-7500F scanning electron microscope (Jeol Ltd., Tokyo, Japan). The samples were first cooled down in liquid nitrogen under glass transition temperature and then fractured into small fragments with surface area of 3 × 2 mm. The fractured surface was covered with a thin layer of gold and put into the microscope.

## 3. Results and Discussion

### 3.1. Influence of Ferrite on Physical-Mechanical and Shielding Characteristics of Composites

In the first part of the study, manganese-zinc ferrite was incorporated into the matrix based on NBR in the amount from 100 phr to 500 phr. The rubber composites were cured at 160 °C using sulfur based curing system. First, electromagnetic parameters and absorption shielding characteristics were investigated. As already outlined, generally used electronic devices emit electromagnetic radiation at low frequencies (0.6–3 GHz). Therefore, the composites used as efficient EMI shields should have absorption maxima within this frequency range. The absorption shielding efficiency of composites was investigated over the frequency range of 1 MHz–3 GHz.

**Figure 1:**
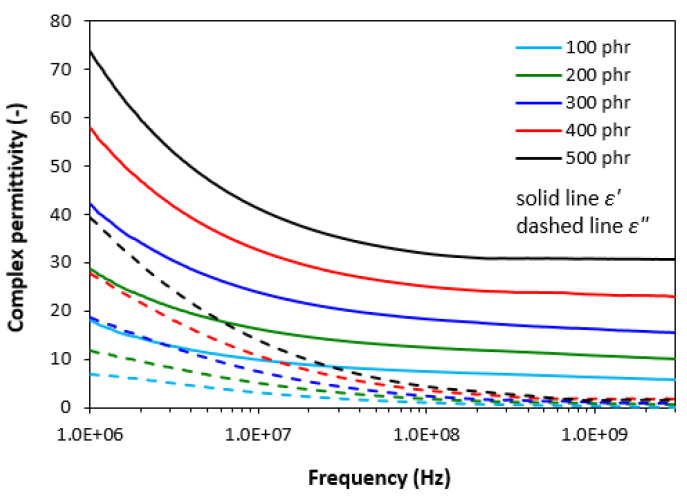
Frequency dependences of real ε′ and imaginary ε″ parts of complex permittivity for composites filled with ferrite.

The frequency dependencies of real ε′ and imaginary ε″ parts of complex (relative) permittivity ε = ε′ − jε″ for the composite materials are graphically illustrated in Figure 1. It becomes obvious that the real part ε′ steeply decreases at frequencies up to about 10 MHz, then settles on a constant value. The initial decline of real permeability can be attributed to the semiconductive nature of manganese-zinc ferrite. It can also be seen that with increasing content of ferrite in composites, the real permittivity shifts to higher values. When the amount of magnetic filler increased from 100 to 500 phr, the real permittivity increased from 18.2 to 73.8. With increasing frequency of electromagnetic radiation, the differences in real permittivity became less visible. From frequency dependences of imaginary permittivity it is possible to observe the similar decreasing trend of ε″. The real permittivity of composites provides higher values when compared to equivalent imaginary permittivity and the differences in ε″ values became negligible at frequencies above 1 GHz. The achieved changes in frequency dependencies of complex permittivity may be attributed to various types of polarization mechanisms originating in the filler as well as the rubber matrix owing to their dielectric character (mainly the interfacial polarization caused by space charges, which accumulate at boundaries of the filler-rubber interface).

The absorption shielding effectiveness of composite materials was investigated through the determination of return loss in decibels unit. The return loss provides valuable information about the amount of electromagnetic radiation, which can be absorbed by the shield. It has been reported in scientific studies that materials reaching return loss at −10 dB can effectively absorb 95% of incident electromagnetic radiation. The return loss at −20 dB is equivalent with 99% absorption of EMI [20,21].

**Figure 2:**
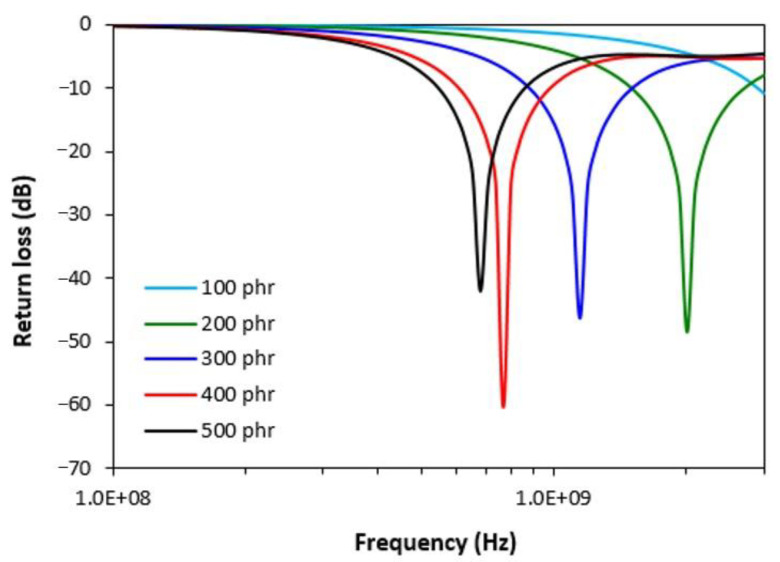
Frequency dependences of return loss for composites filled with ferrite.

Figure 2 depicts the return loss of composites with different contents of ferrite in the tested frequency range. It is shown that all composites containing 200 phr of filler and more provide satisfactory absorption shielding efficiency. It also becomes apparent that with increasing content of magnetic filler, the absorption maxima and absorption shielding efficiency of composites shift to lower frequencies of electromagnetic radiation. The composite containing 200 phr of manganese-zinc ferrite exhibited return loss at −10 dB and −20 dB in widest frequency range, i.e., from 1.5 GHz to 2.7 GHz at −10 dB and from 1.85 GHz to 2.2 GHz at −20 dB. This composite can be considered to be the best absorption shielding material. The absorption maximum of this composite shield is at −48 dB at a frequency of 2 GHz of the incident electromagnetic radiation. The composite filled with 400 phr of ferrite showed a lower absorption maximum (−60 dB), but also a lower frequency range, in which it is able to effectively absorb EMI (return loss at −10 dB in the frequency range 0.6–0.98 GHz and return loss at −20 dB in the frequency range only 0.72–0.83 GHz). The composite containing 500 phr of ferrite was found to have the highest absorption maximum and the lowest effective absorption frequency range. The selected electromagnetic absorption parameters as the minimum value of return loss *RL*_min_ at a matching frequency f_m_, matching frequency f_m_, bandwidth Δf for return loss at −10 and at −20 dB, are presented in Table 5. Based upon the achieved results it can be stated that the tested composites can be used for EMI shielding applications at frequencies over 500 MHz.

**Figure 3:**
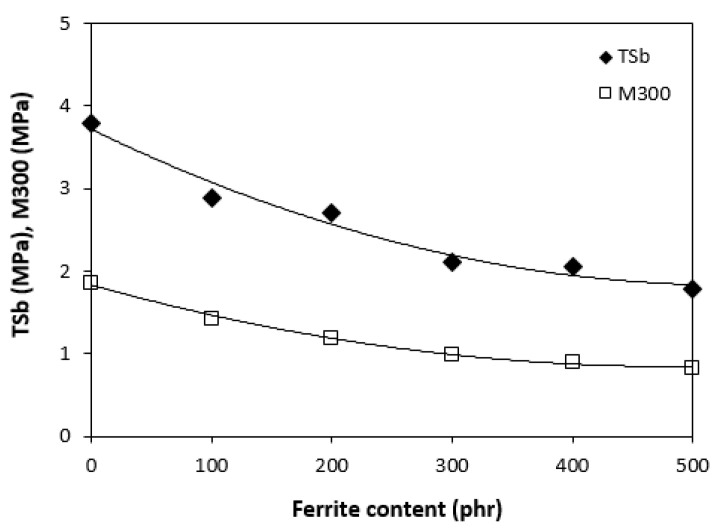
Influence of ferrite content on modulus M300 and tensile strength of composites.

**Figure 4:**
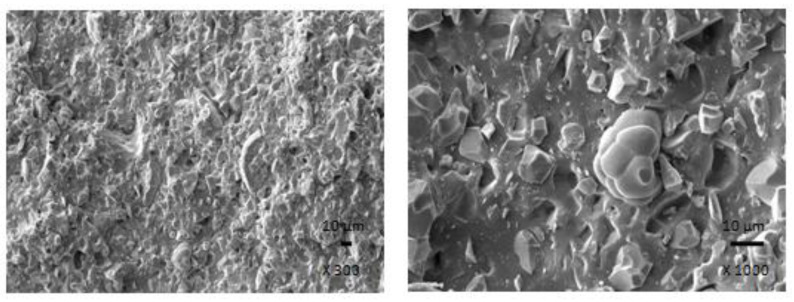
SEM images of composite filled with 300 phr of ferrite.

The results obtained from determination of physical-mechanical properties revealed that modulus at 300% elongation (M300) and tensile strength of composites were found to decrease with increasing content of magnetic filler (Figure 3). It becomes evident that manganese zinc ferrite behaves as inactive filler in the rubber matrix. The reason can be attributed to the high level of filler loading and poor compatibility and adhesion between the rubber and the filler on their interface. SEM analysis confirmed the presumption and revealed that the adhesion between the filler and the rubber is weak with existence of voids and cavities on the filler-rubber interface (Figure 4). Those in-homogeneities and voids were likely caused by fracturing of composite samples for their preparation for SEM analysis and dropping out poorly bonded ferrite particles. The results of the research revealed that application of magnetic soft manganese-zinc ferrite into the NBR-based matrix leads to the preparation of rubber magnetic composites with the effect of EMI shielding. The main advantage of these composites is their ability to shield electromagnetic waves by absorption mechanisms. On the other hand, physical-mechanical properties, like tensile strength and moduli were deteriorated, which is a negative aspect from the point of practical industrial applications.

### 3.2. Influence of Carbon Black on Physical-Mechanical and Shielding Characteristics of Composites

**Figure 5:**
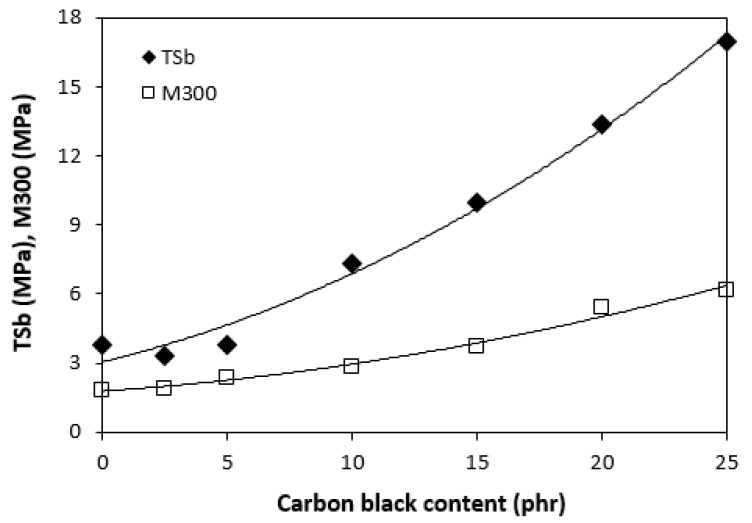
Influence of CB on modulus M300 and tensile strength of composites.

**Figure 6:**
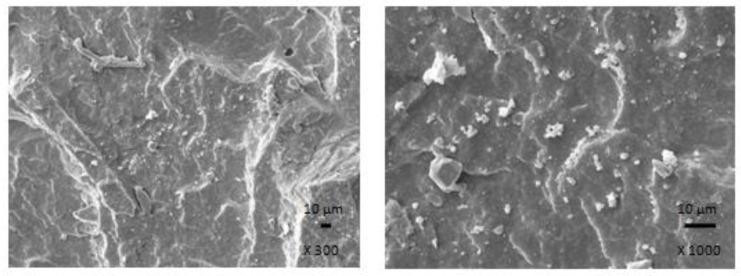
SEM images of composites filled with 20 phr of CB.

In the following study, rubber composites were fabricated by incorporation of carbon black into NBR based matrix in concentration scale ranging from 2.5 phr to 25 phr and subsequently cured at 160 °C. The physical-mechanical properties were investigated and the influence of CB content on modulus M300 and tensile strength is presented in Figure 5. As seen, both characteristics exhibited increasing trend with increasing content of CB. The tensile strength increased from less than 4 MPa for the reference to over 17 MPa for the maximally filled composite. Based upon the achieved results it becomes clearly apparent that CB reinforces the rubber matrix. In generally, the nature of the reinforcing effects of fillers in the rubber matrix lies in mutual interactions between the rubber and the filler on the filler-rubber interface, where physical or chemical couplings between both components are formed. Thus, good adhesion and compatibility between the rubber and the filler are the most important factors for the reinforcement of rubber composites. From Figure 6 it is possible to observe that homogeneity and mutual adhesion between carbon black and rubber is much higher when compared to that of ferrite-rubber (Figure 4). The reason can be attributed to much lower dimensions of CB particles and strong physical or even physical-chemical interactions between the two components.

**Figure 7:**
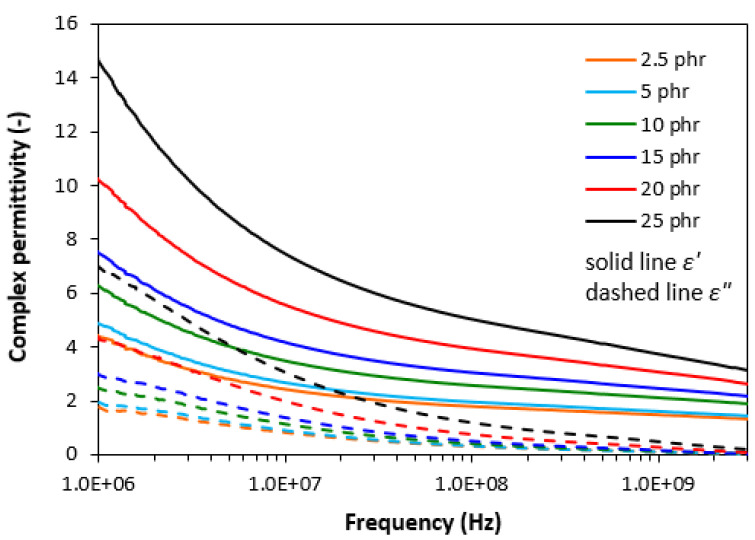
Frequency dependences of real ε′ and imaginary ε″ parts of complex permittivity for composites filled with CB.

**Figure 8:**
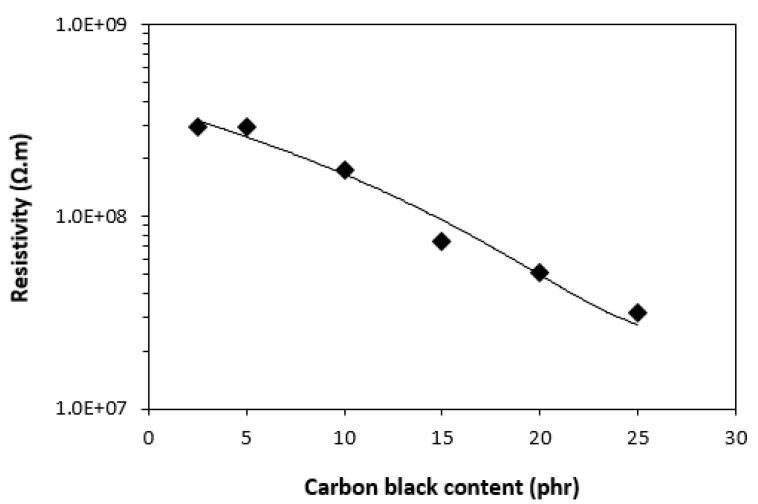
Influence of CB on resistivity of composites.

From frequency dependences of complex permittivity for CB filled composites it can be seen that both, the real ε′ and imaginary ε″ parts decreased with increase in frequency (Figure 7). It becomes also evident that both parts were dependent on the content of CB, mainly at low frequencies. With increasing frequency of electromagnetic radiation, the differences in permittivity became smaller. The frequency dependences of permittivity are not only influenced by polarization mechanisms (mainly interfacial polarization) but also by resistivity of electrically conductive carbon black. As seen in Figure 8, the resistivity of composites decreased with increasing content of CB owing to good electrical conductivity of carbon black. By increasing the content of CB from 2.5 phr to 25 phr, the resistivity of composites decreased by almost one order of magnitude, from 2.9 × 10^8^ Ω∙m for the composite with 2.5 phr of CB down to 3.2 × 10^7^ Ω∙m for the maximally filled composite.

**Figure 9:**
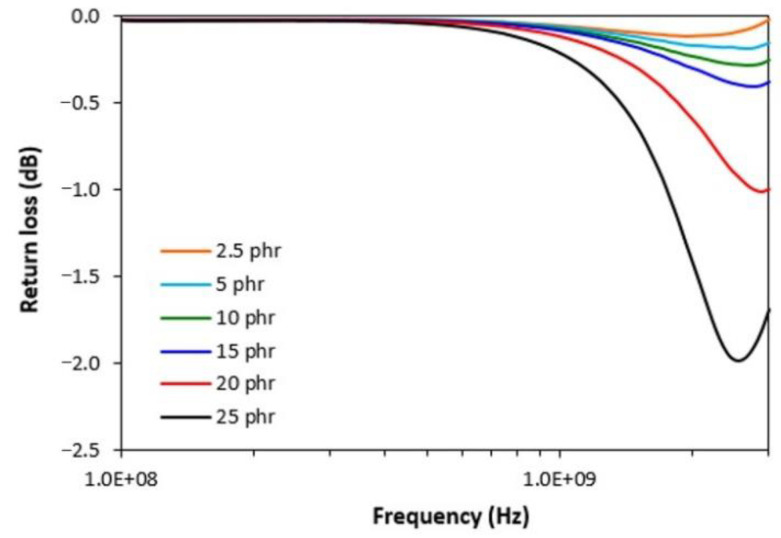
Frequency dependences of return loss for composites filled with CB.

From Figure 9 it is shown that the absorption shielding efficiency of composites slightly increases with increasing content of carbon black. The lowest value of return loss reached the composite filled with 25 phr of CB. However, the absorption maximum is at only −2 dB, which is not sufficient to reach satisfactory absorption shielding efficiency.

The results of the study revealed that carbon black reinforces the rubber matrix and contributes to the improvement of physical-mechanical properties and electrical conductivity of composites. However, those composites are not able to absorb EMI in the tested frequency range. The main reason can be attributed to the high electrical conductivity of CB, which is very important criterion for reflection shielding.

### 3.3. Influence of Combination of Carbon Black and Ferrite on Physical-Mechanical and Shielding Characteristics of Composites

In the third part of the study, carbon black was combined with manganese-zinc ferrite in order to prepare rubber magnetic composites. The amount of CB was kept constant in all composites −20 phr, while magnetic filler was dosed to rubber composites in concentration scale ranging from 100 to 500 phr.

**Figure 10:**
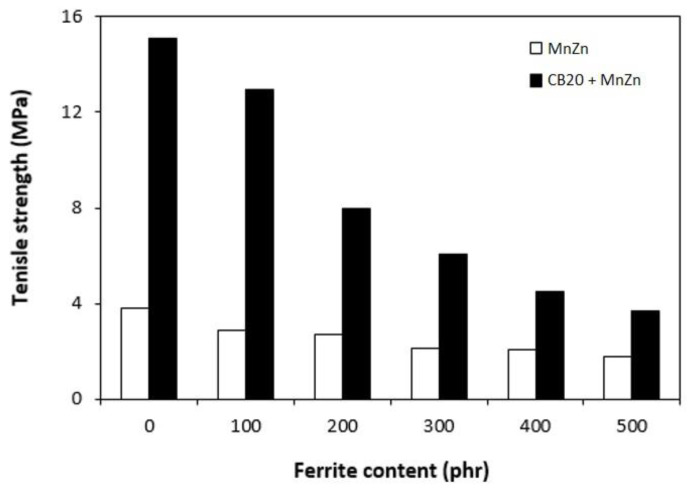
Influence of ferrite content on modulus M300 of hybrid CB/ferrite composites.

**Figure 11:**
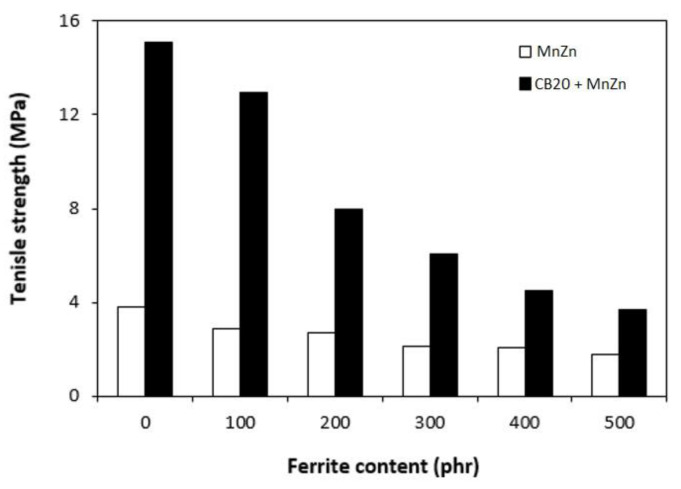
Influence of ferrite content on tensile strength of hybrid CB/ferrite composites.

**Figure 12:**
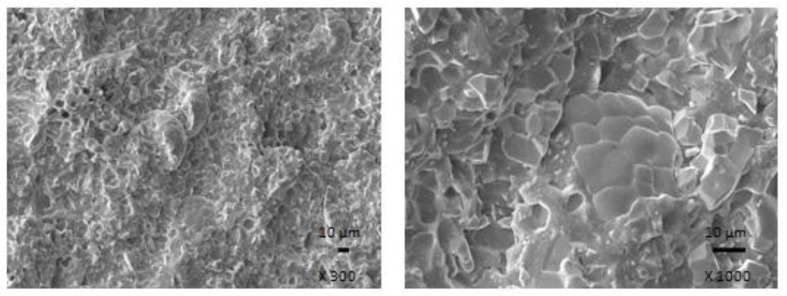
SEM images of composite filled with 20 phr of CB and 300 phr of ferrite.

The physical-mechanical properties of composites filled with combination of CB and ferrite are, in graphical illustrations, compared with the characteristics of equivalent composites filled only with magnetic filler. As shown in Figure 10, modulus M300 of hybrid CB/ferrite composites was higher in comparison with the corresponding composites filled with magnetic filler. Similarly, composites filled only with ferrite exhibited lower tensile strength (Figure 11). The application of CB leads to the reinforcement of the rubber matrix and thus to the increase of tensile strength. The greatest difference in tensile strength was possible to see in the case of reference samples and composites with lower ferrite content. With increasing amount of magnetic filler, the difference in tensile strength of tested composites became less visible, but the tensile strength of hybrid CB/ferrite composites was still higher compared to those filled with magnetic soft ferrite. SEM analysis of hybrid composites (Figure 12) demonstrated that the dispersion of fillers, mainly ferrite in hybrid composites was higher when compared to ferrite filled composites (Figure 4). This can be attributed to the higher viscosity of the rubber matrix due to the presence of CB and, thus, higher shear stress during compounding, which facilitates the dispersion and distribution of ferrite particles within the rubber matrix. Higher dispersion state of ferrite in the rubber matrix might also contribute to better physical-mechanical properties of hybrid composites.

**Figure 13:**
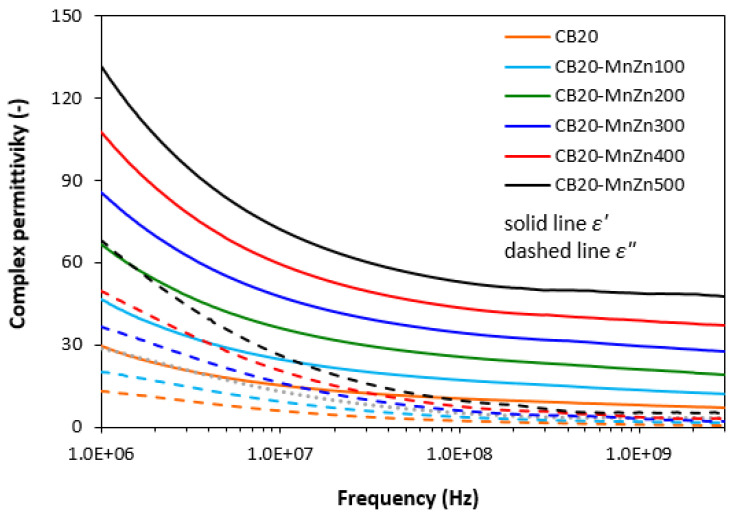
Frequency dependences of real ε′ and imaginary ε″ parts of complex permittivity for hybrid CB/ferrite composites.

**Figure 14:**
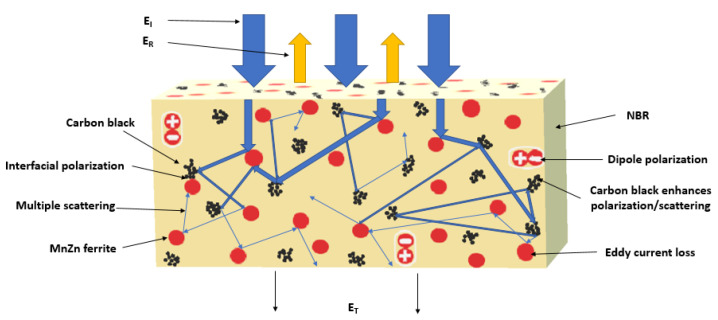
Schematic illustration of EMI shielding mechanisms for hybrid CB/ferrite composites.

Looking at Figure 13, one can see very similar behavior of real ε′ and imaginary ε″ parts of complex permittivity for hybrid composites in dependence on frequency and fillers content as in the case of equivalent composites filled only with ferrite (Figure 1) or carbon black (Figure 7). As seen, after sharp decrease of ε′ at frequencies up to about 10 MHz, it fluctuates in a low range of experimental values. The lowest ε′ was found to have the composite filled with 20 phr of CB (ε′ = 30 at 1 MHz). By increasing frequency up to 3 GHz, it decreased to 7. The real permittivity of the hybrid composite filled with 20 phr of CB and 100 phr of ferrite decreased from 47 down to 12 when the electromagnetic radiation frequency increased from 1 MHz to 3 GHz. The increase in magnetic filler content to 500 phr resulted in the increase of real permittivity up to almost 132 at 1 MHz. Then, it dropped down to 48 at the maximum tested frequency. The similar trend can be also observed in frequency dependencies of imaginary permittivity. The imaginary part ε″ is lower in comparison with real part ε′ of corresponding composites in the whole tested frequency range. The imaginary permittivity of the maximally filled hybrid composite was reduced from 68 at 1 MHz to 5 at 3 GHz. The initial decrease of real permittivity with frequency may be attributed to the semiconductive character of manganese-zinc ferrite and conductive character of carbon black. The increasing amount of filler loading results in the increase of both, real and imaginary permittivity. As also shown, the real and imaginary permittivity of hybrid CB/ferrite composites are much higher when compared to corresponding composites filled only with ferrite or carbon black. The real permittivity is related to the electrical charge storage and mainly associated with the amount of polarization in the material, whereas the imaginary part is related to the loss in energy (dielectric loss). Polarization of the filler, rubber matrix as well as interfacial polarization can occur in dependence on frequency range [22,23]. The dielectric loss can be attributed to interfacial polarization, dipole and electronic polarization, natural resonance, and relaxation phenomena [24,25]. The presence of a high amount of interfaces in the composites paves the way for interfacial polarization, which occurs on the CB particles with relatively high conductivity. This leads to the accumulation of charges at interfaces and generation of dipoles on semiconductive ferrite particles (Figure 14). Thus, the interfacial polarization and associated space charge relaxation processes contribute to the EMI shielding performance [26]. In hybrid CB/ferrite rubber composites, manganese-zinc ferrite acts as a polarized center in the presence of electromagnetic radiation, which provides space for better absorption shielding.

**Figure 15:**
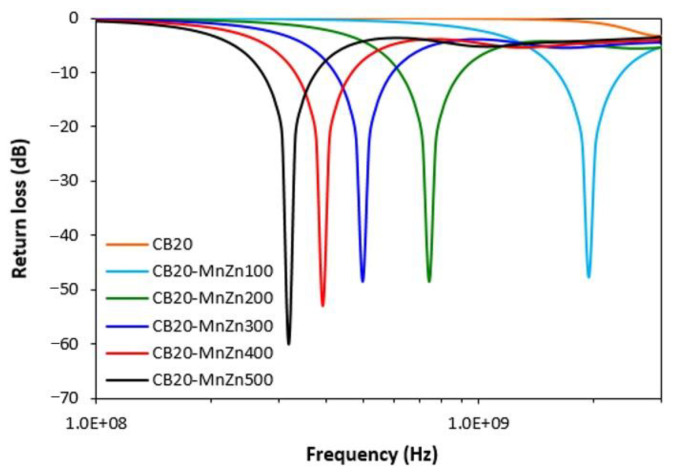
Frequency dependences of return loss for hybrid CB/ferrite composites.

From the graphical illustration of the frequency dependence of the return loss for hybrid composites (Figure 15) it is shown that with exclusion of the composite filled only with CB, all composites containing manganese-zinc ferrite exhibited absorption shielding ability. The best absorption shielding material can be considered the composite containing 100 phr of manganese-zinc ferrite, because this material has a return loss at −10 dB in the widest frequency range, i.e., from 1.6 to 2.35 GHz. The absorption maximum is at −48 dB at 1.9 GHz of the incident electromagnetic radiation. The lowest value of return loss reached the composite filled with 500 phr of ferrite (−60 dB). Although, this composite also exhibited the lowest efficient absorption bandwidth at −10 and −20 dB, as seen in Table 6. It becomes apparent from Figure 15 and Table 6 that the increasing content of magnetic filler in hybrid composites resulted in lower absorption maxima and the total absorption shielding efficiency of composites shift to lower frequencies. Additionally, with the increase in the magnetic filler content, the efficient absorption frequency ranges of composites become narrower. The computed values of electromagnetic absorption parameters, summarized in Table 6, indicate that the prepared composite materials can be thought as suitable candidates for EMI shielding applications at frequencies above 300 MHz. When comparing composites filled only with ferrite (Figure 2, Table 5) and composites filled with a combination of CB and ferrite (Figure 15, Table 6), it becomes obvious that hybrid composites demonstrate lower absorption maxima (lower values of return loss), but also lower matching frequencies and narrower bandwidth for return loss at −10 dB and −20 dB. This means that the combination of ferrite with carbon black resulted in the shifting of efficient absorption shielding ability to lower frequencies of electromagnetic radiation on one hand. On the other hand, based upon narrower absorption peaks of hybrid composites, it can be stated that absorption shielding ability of those composites is lower when compared to equivalent composites filled only with ferrite. The reason can be attributed to the increasing of electrical conductivity and complex permittivity for hybrid composites due to the presence of conductive carbon black. As outlined, the materials with high conductivity are more prone to EMI shielding with dominant reflection mechanism, especially at low frequencies [27,28]. Another factor that might also contribute to the different absorption shielding behavior could be different dispersion and distribution of ferrite in composites filled only with magnetic filler (Figure 4) and hybrid CB/ferrite composites (Figure 12).

## 4. Conclusions

This work was aimed at the investigation of manganese-zinc ferrite, CB, and combination of ferrite with CB on physical-mechanical properties and EMI absorption shielding characteristics of composites based on NBR.

The results demonstrated that composites filled with manganese-zinc ferrite can be used as efficient EMI shields with absorption dominated shielding mechanism in the tested frequency range 1 MHz–3 GHz. However, the tensile strength and modulus M300 of composites showed decreasing trend with increasing content of magnetic filler, which suggests that ferrite behaves as an inactive filler. On the other hand, composites filled with CB exhibited applicable physical-mechanical properties, but their absorption shielding performance was insufficient. In hybrid CB/ferrite composites, the presence of CB resulted in better dispersion of ferrite within the rubber matrix and improvement of physical-mechanical properties of composites. On other hand, conductive carbon black contributed to the charge storage, various polarization mechanisms and related relaxation phenomena within the rubber matrix, which was reflected in the increase of real and imaginary permittivity. This was subsequently reflected in the modification of absorption peaks and corresponding electromagnetic absorption parameters for hybrid composites. The best absorption shielding material, among all tested composites, can be considered the solely ferrite-filled composite containing 200 phr of magnetic filler that exhibited the widest absorption frequency bandwidth for return loss at −10 dB and −20 dB, which indicates the absorption of 95% or 99% of EMI, respectively. The results also revealed that the improved tensile characteristics of hybrid CB/ferrite composites could contribute to the extension of application potential field of the EMI shields. The preparation of materials not only with good absorption shielding performance, but also with applicable physical-mechanical features paves the way for the fabrication of composite shields that could be extensively used for the protection of commercial electronic equipment, such as radios, TV sets, computers, mobiles phones, or microwave ovens.

## Figures and Tables

**Table 1:** Characteristics of manganese-zinc ferrite.

Characteristics	Values
Particle size (µm)	0.1–30
Specific surface area (m^2^/g)	10.99
Total porosity (%)	59.72
Density (g/cm^3^)	4.87
Electrical resistivity (Ω∙m)	3

**Table 2:** Composition of composites filled with ferrite.

Component	NBR	ZnO	Stearic Acid	CBS	Sulfur	Ferrite
Content (phr)	100	3	2	1.5	1.5	0–500

**Table 3:** Composition of composites filled with carbon black.

Component	NBR	ZnO	Stearic Acid	CBS	Sulfur	CB
Content (phr)	100	3	2	1.5	1.5	0–25

**Table 4:** Composition of composites filled with combination of ferrite and carbon black.

Component	NBR	ZnO	Stearic Acid	CBS	Sulfur	CB	Ferrite
Content (phr)	100	3	2	1.5	1.5	20	0–500

**Table 5:** Electromagnetic absorption parameters for composites filled with ferrite.

Content of Ferrite (phr)	*RL*_min_ (dB)	f_m_ (MHz)	Δf (MHz) for *RL* at −10 dB	Δf (MHz) for *RL* at −20 dB
100	—	—	—	—
200	−48	2010	1220	370
300	−46	1148	630	188
400	−60	769	380	115
500	−42	682	310	103

**Table 6:** Electromagnetic absorption parameters for hybrid CB/ferrite composites.

Content of Ferrite (phr)	*RL*_min_ (dB)	f_m_ (MHz)	Δf (MHz) for *RL* at −10 dB	Δf (MHz) for *RL* at −20 dB
100	−48	1931	750	158
200	−49	739	270	73
300	−49	495	170	54
400	−53	390	140	42
500	−60	319	110	21

## Data Availability

Data is contained within the article.
